# Socially Aware Heterogeneous Wireless Networks

**DOI:** 10.3390/s150613705

**Published:** 2015-06-11

**Authors:** Pavlos Kosmides, Evgenia Adamopoulou, Konstantinos Demestichas, Michael Theologou, Miltiades Anagnostou, Angelos Rouskas

**Affiliations:** 1 School of Electrical and Computer Engineering, National Technical University of Athens, Athens 15773, Greece; E-Mails: eadam@cn.ntua.gr (E.A.); cdemest@cn.ntua.gr (K.D.); theolog@cn.ntua.gr (M.T.); miltos@cn.ntua.gr (M.A.); 2 Department of Digital Systems, University of Piraeus, Piraeus 18534, Greece; E-Mail: arouskas@unipi.gr

**Keywords:** heterogeneous wireless networks, software defined networks, software-based controllers, social networks, learning algorithms, mobile operator recommendations

## Abstract

The development of smart cities has been the epicentre of many researchers’ efforts during the past decade. One of the key requirements for smart city networks is mobility and this is the reason stable, reliable and high-quality wireless communications are needed in order to connect people and devices. Most research efforts so far, have used different kinds of wireless and sensor networks, making interoperability rather difficult to accomplish in smart cities. One common solution proposed in the recent literature is the use of software defined networks (SDNs), in order to enhance interoperability among the various heterogeneous wireless networks. In addition, SDNs can take advantage of the data retrieved from available sensors and use them as part of the intelligent decision making process contacted during the resource allocation procedure. In this paper, we propose an architecture combining heterogeneous wireless networks with social networks using SDNs. Specifically, we exploit the information retrieved from location based social networks regarding users’ locations and we attempt to predict areas that will be crowded by using specially-designed machine learning techniques. By recognizing possible crowded areas, we can provide mobile operators with recommendations about areas requiring datacell activation or deactivation.

## 1. Introduction

During the past few decades, wireless networks have been developing at high speed, experiencing numerous phases of evolution like 2G and 3G to the recent 4G networks. This evolution has resulted in the presence of numerous radio access technologies (RATs) with diverse network characteristics [1,2]. In parallel, an explosive growth in mobile phone (e.g., smartphone) use has occurred, and by 2020 the traffic originating from portable devices is expected to increment exponentially [3]. The anticipation of the aforementioned increase is also strengthened by the extension of mobile data communication usage to machines, sensors and smart objects, which are major contributors for the development of smart cities. These heterogeneous wireless devices will be connected to each other, requiring low latency and extremely high reliability. In order to cope with this change in interconnected mobile device patterns, the research and industrial communities have lately focussed their efforts on the advancement of wireless communication technologies, by developing the next generation (5G) of wireless networks [4]. A significant challenge emerging from this effort is the simultaneous operation of distinctive RATs in a heterogeneous wireless environment.

A recent approach towards the convergence of heterogeneous wireless networks is the use of software defined networks (SDNs). One of the main objectives of SDNs, is to centralize the network intelligence in software-based controllers [5]. The application of this technology enables mobile operators to enhance their decision making process for resource allocation issues. One of the major issues that mobile operators are confronted with is the activation and deactivation of the available datacells. By doing so, they can achieve significant energy and economic savings while improving the overall quality of service (QoS) [6,7].

However, no specific effort on introducing social network awareness to SDNs has been yet recorded. In this paper, we propose the combination of SDNs with online social networks (OSNs). Specifically, we propose an innovative system architecture that takes advantage of software-based controllers and imports social awareness from OSNs, in order to assist the centralized controller in the decision making process, by applying machine learning techniques. In order to provide recommendations to mobile operators about areas where they can activate or deactivate datacells, we propose the use of three different learning algorithms, and provide predictions about the crowd level that is expected in specific sub-areas. In addition, in order to reduce the required resources, we propose the clustering of the available sub-areas to group-areas that exhibit similar characteristics. The proposed solution is agnostic of the used wireless access technology. Its purpose is to complement the mobile operators’ collected data, while it can, also, be applied in different density levels; for example by activating appropriate picocells inside macrocells.

The rest of this paper is organized as follows: in Section 2 an overview of the relevant research literature is presented; Section 3 describes the proposed systems’ architecture, while the designed architecture for the implementation of the abovementioned service is described in detail, including foreseen components and the specified interactions among them. The architecture is defined using a well-known modelling language, namely *ArchiMate*^®^ [8], showing the main components and the relationships among them.; in Section 4, three different approaches used based on machine learning are presented, namely multilayer perceptrons (MLP), support vector machines (SVM) and probabilistic neural networks (PNN). The use of the described learning techniques per geographical location area (sub-area), as well as per cluster of sub-areas (group-area) following similar patterns is proposed. The dataset used is presented in Section 5, while in Section 6 we provide the acquired results and discuss the performance of the proposed algorithms. Two indicative use case scenarios are presented in Section 7 while Section 8 concludes the paper.

## 2. Related Work

### 2.1. Next Generation Wireless Networks

The use of SDNs as one of the most promising technologies to meet the challenges arising from the development of the next generation wireless networks is proposed in several research efforts. In this context, the authors in [9] propose the utilization of software defined radio access networks (SoftRANs), a software defined centralized control plane for radio access networks, and present a preparatory outline and architecture along with use cases and a feasibility analysis. Similarly in [10], the authors present OpenRAN, an architecture for software-defined RAN via virtualization.

In [11] the authors propose a centralized SDN controller, *i.e.*, a software-defined RAN (SD-RAN), which abstracts all the resources made accessible by a pool of base stations (BSs) into a solitary large resource pool, namely the virtual cell (V-Cell). Finally, in [12] the authors state that SDNs can simplify the design and management of cellular data networks, while enabling the introduction of new services. To this end, they propose extensions to existing controller platforms, switches and BSs to enable software defined cellular networks.

Another technology that emerged during the last decade is social computing. Specifically, in recent years the use of social networks (SNs) has increased rapidly with an escalating acceptance by the users. Moreover, recent efforts concentrate on taking advantage of the knowledge extracted from SNs and confront problems in wireless communications. In particular, the authors in [13] present a social-connectivity-aware vertical handover (SCVH) scheme, which uses connectivity graph data from OSNs in order to perform admission control in wireless local area networks (WLANs). Similarly in [14], the authors take into account the social interactions among users and present a methodology to deal with network disruption in opportunistic networking solutions.

A framework for addressing interplay between OSNs and wireless communications is presented in [15]. The authors apply utility-based engineering theory principles for resource management by adopting elements from social network analysis. Finally, the authors in [16] recognize the dependence of social relationships and behaviours on users’ movements and the importance of social awareness for the design of networking solutions. Specifically, they present a survey for the socially aware networking field, focusing on three aspects, namely routing and forwarding, incentive mechanisms and data dissemination. In this paper, following the above trends, we propose the use of social networks in order to enhance the intelligence of SDN solutions. Specifically, we introduce a service entity that can be applied on the SDNs architectures, during the development of future 5G networks.

### 2.2. Predicting Crowded Areas

Another issue discussed in the literature is the prediction of crowded areas using learning algorithms. Similar to this approach, the authors in [17] recognize the benefits that can be acquired from predicting user future location and apply location prediction to wireless cellular networks, by estimating the expected number of users in different parts of the network, with granularity of a BS. In [18] the authors identify the impact of crowded events to the performance of voice and data services provided by cellular network operators. They illustrate how changes in population distribution, user behaviour, and application workload during crowded events result in significant voice and data performance degradation. Similarly, in [19] the authors deal with the analysis of crowd mobility during special events. They support the view, that the prediction of the arrival of people in future events is feasible, due to the observation that events of the same type show similar spatial distribution.

Finally in [20] the authors combine the information collected from cellular data, with a dataset of geo-tagged venues from Foursquare [21] and formulate the problem of urban activity inference in a supervised learning framework. In particular, they analyse the check-in patterns of Foursquare users at places geographically close to BSs and exploit user communication patterns observed at these BSs in order to predict the activity of Foursquare users who check-in at nearby venues.

The main goal of the architecture proposed in this paper is to enhance the decision making process conducted by mobile operators, by providing recommendations about whether to activate or deactivate datacells in specific sub-areas. To achieve this, in accordance to the aforementioned research efforts, we propose the use of machine learning techniques in order to make predictions about the expected crowd level.

## 3. System Description

**Figure 1:**
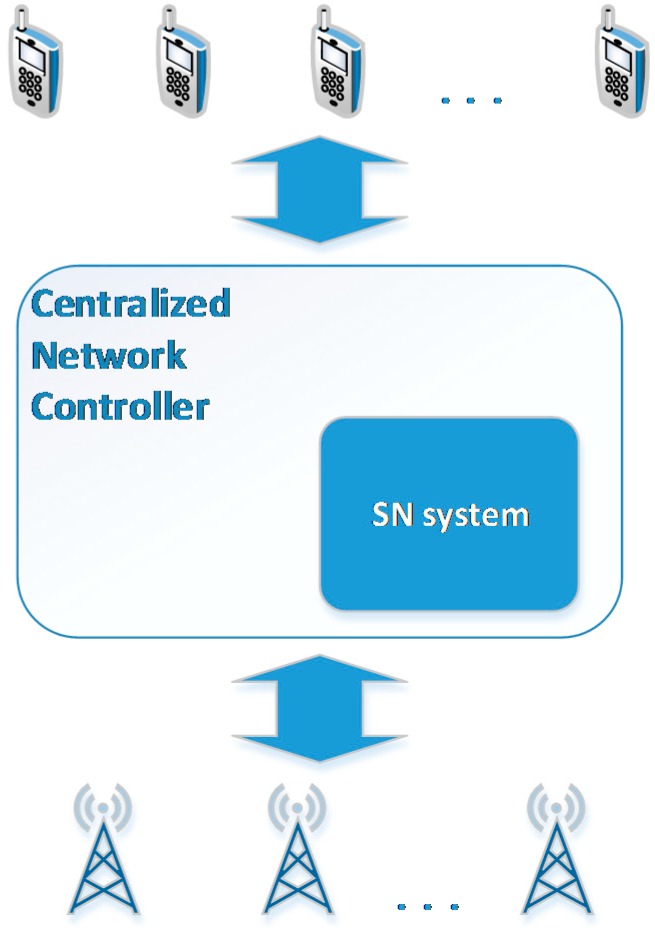
SDN-based system’s architecture.

In this paper we rely on the architecture presented in [9,10], which is based on the use of SDNs and enables the abstraction of low level networking functionality by means of virtual services, allowing the introduction of new services [4]. A representation of the entities involved in the scope of this paper is depicted in Figure 1. We assume a *Centralized Network Controller*, based on the SDN architecture, which is responsible for collecting all necessary measurements and reaching the required decisions. In order to enhance the *Centralized Network Controller* with information extracted from social networks, the *SN system* entity is introduced.

**Figure 2:**
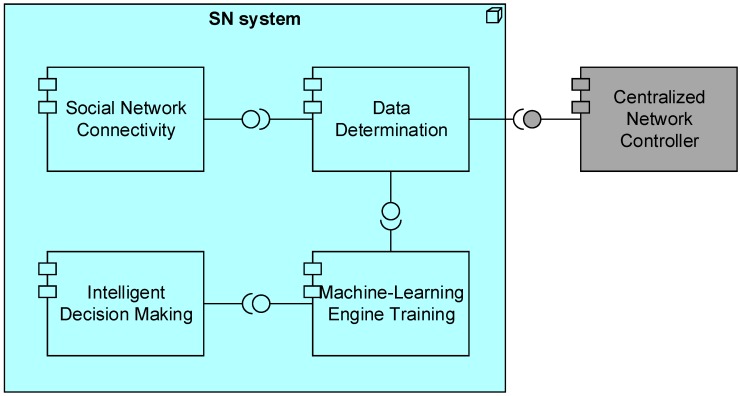
SN system—technology layer (deployment architecture).

The *SN system* entity is responsible for connecting to SN services and for crawling data that are related to users of the specific geographical area covered by the controller. In addition, using machine learning mechanisms we can assist the *Centralized Network Controller* with the decision making process. In Figure 2, the deployment of the main application components of the proposed architecture is illustrated, using the *ArchiMate*^®^ notation [8]. The *SN system* is composed of four application components, and it is connected to the *Centralized Network Controller* component. A more thorough analysis of the main components is presented below.

*Social Network Connectivity*—This component is responsible for establishing the connections of the *Centralized Network Controller* with available SNs. It uses the available APIs provided from each SN and after the application of the required policies addressed by the SNs services, it links the *Centralized Network Controller* with the SN.

*Data Determination*—This component is responsible for collecting information from both BSs and connected SNs. From the BSs, details like the physical location and each BS’s range are defined, while the crawling of data from SNs is limited to the users that are located in each BS’s range.

*Machine-Learning Engine Training*—This component is responsible for the centralized training of machine-learning engines which will be used by *Intelligent Decision Making* component.

*Intelligent Decision Making*—This component is responsible for taking into account the current status of the *Centralized Network Controlle*r and by using the trained machine learning engines (MLEs) reaches decisions regarding resource allocation, energy efficiency, *etc*.

We assume that the necessary data from social networks have already been crawled. The goal of this paper is to study the performance of machine learning techniques in order to provide recommendations to operators about whether to activate or deactivate datacells in specific sub-areas. Specifically, we propose the use of three different learning algorithms in order to make predictions about the expected crowd level.

## 4. Prediction Models

In order to provide mobile operators with recommendations about potential sub-areas where datacell activation or deactivation can be applied, we propose the use of machine learning techniques in order to predict areas that are expected to be crowded, based on input retrieved from location-based SNs.

### 4.1. Prediction per Area

In order to decide how many datacells will be active, we separate each geographical area covered by the *Centralized Network Controller* into sub-areas, with each sub-area possibly consisting of more than one BS. For each sub-area, we create a separate MLE, in which we apply a prediction model, in order to predict the number of the expected active users. Three different prediction algorithms based on machine learning, namely MLP, SVM and PNN, are used. The purpose of the following subsections, is not to give a detailed description of the prediction models, but rather to provide the reader with the necessary background information so as to follow up the proposed scheme.

#### 4.1.1. Multilayer Perceptron

The first approach used as a prediction model is the MLP network. MLPs form one type of feed-forward Artificial Neural Networks (ANNs) according to the taxonomy of neural network architectures presented in [22,23].

ANNs, inspired by the human brain learning system, are among the most effective machine learning methods currently utilized. Their objective is to approximate a target function using a training set of input and output data. One major advantage of ANNs is their robustness to errors occurring in the training data [24]. The basic unit element of neural networks is the neuron which consists of three elements:

- A set of connecting links, called synapses, each characterized by a weight $w_{kj}$, which is the *j* input of the *k* neuron.
- An adder for summing the input signals $x=\left[x_{1},x_{2},\ldots ,x_{n}\right]$.
- An activation function φ(·) that defines the output of the neuron.

An externally applied bias $b_{k}$ is also used to increase or decrease the input of the activation function. The output $u_{k}$ of the summation unit of the neuron *k* is given as:

(1) $$u_{k}=\;\overset{n}{\underset{j=1}{\sum }}w_{kj}x_{j}$$

and the output $y_{k}$ of the neuron k is expressed as:

(2) $$y_{k}=\;\text{φ}\left(u_{k}+b_{k}\right)$$

As mentioned above, a common implementation of ANNs is the MLP, which consists of one input layer, one output layer and one or more hidden layers. The main characteristic of an MLP network is that the neurons of each layer are fully connected to the neurons of the following layer. An important feature in the construction of an MLP is the number of neurons used in the hidden layer. A small number of neurons in the hidden layer can lead to underfitting, while a large number of neurons can lead to overfitting, resulting in poor performance over new unseen data. As a result, there is a trade-off concerning the choice of the number of hidden layer neurons. It is argued that any function can be approximated to arbitrary accuracy by a three-layer neural network [24,25].

#### 4.1.2. Support Vector Machine (SVM)

Another category of universal feed-forward network is the SVMs, proposed by Vapnik [26,27,28], which are widely used for pattern classification and non-linear regression problems. This approach is based on statistical learning theory and uses linear, polynomial and radial basis kernels. An SVM, unlike common ANNs, is characterized by the absence of local minima. In addition, the computational complexity of SVMs does not depend on the dimensionality of the input space [29].

Assuming that separable patterns in the context of pattern classification exist, the main idea of SVMs is to construct a hyperplane as the decision surface in such a way that the margin of separation between positive and negative examples is minimized. A major advantage of SVMs is that they can provide a good generalization performance on pattern classification problems despite the fact that they do not incorporate problem-domain knowledge.

A central notion for the construction of the SVM learning algorithm is the inner-product kernel between a “support vector” $x_{i}$ and the vector x drawn from the input space. The support vectors consist of a small subset of the training data extracted by the algorithm. Depending on how this inner-product kernel is generated, different learning machines characterized by nonlinear decision surfaces can be constructed.

#### 4.1.3. Probabilistic Neural Network (PNN)

The PNN is a feedforward ANN, firstly introduced by Specht [30]. PNN is a supervised neural network and is used to perform classification where the target variable is categorical. Compared to the MLP networks, a PNN is usually much faster to train and more accurate. This is mainly due to the fact that the PNN is closely related to Bayes classification rule [30], and Parzen nonparametric probability density function estimation theory [31].

**Figure 3:**
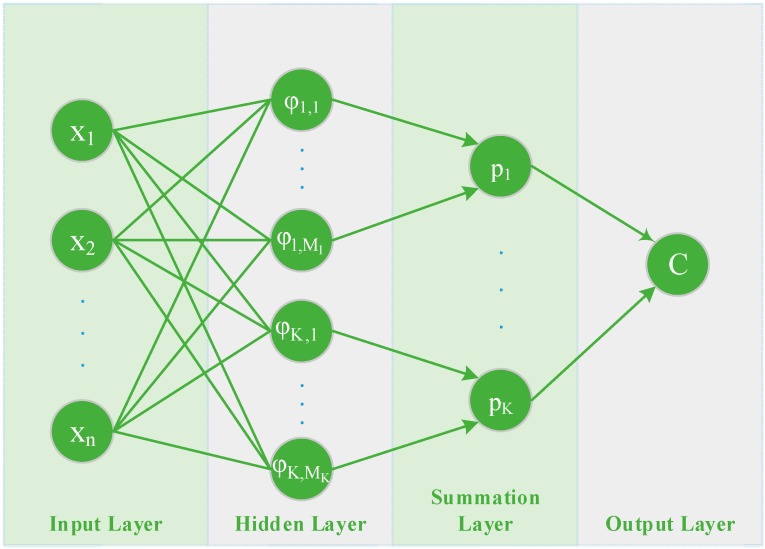
Architecture of a Probabilistic Neural Network.

As can be seen in Figure 3, the architecture of a PNN consists of four layers; the *input* layer, the *hidden* layer, the *summation* layer, and the *output* layer. An input vector $x={\left(x_{1},\;\ldots ,\;x_{n}\right)}^{T}\in {\mathbb{R}}^{n}$, is applied to the n input neurons. The *input* layer does not perform any computation and simply distributes the input to the neurons in the *pattern* layer dividing them into K groups, one for each class. On receiving a pattern x from the *input* layer, the neuron $x_{ij}$ of the *pattern* layer computes its output using a Gaussian kernel of the form:

(3) $$\varphi_{k,i}\left(x\right)=\;\frac{1}{{\left(2\pi \sigma^{2}\right)}^{n/2}}exp\left(-\frac{{\left\|x-x_{k,i}\right\|}^{2}}{2\sigma^{2}}\right)$$

where $x_{k,i}\in {\mathbb{R}}^{n}$ is the centre of the kernel, and σ, also known as the spread (smoothing) parameter, determines the size of the receptive field of the kernel. The *summation* layer is responsible for summing the output of the *hidden* layer and produces a vector of probabilities that represent the probability of each feature that belongs to a specific class, through a combination of the previously computed densities:

(4) $$p_{k}\left(x\right)=\;\overset{M_{k}}{\underset{i=1}{\sum }}w_{ki}\varphi_{k,i}\left(x\right),\;\;k\in \left\{1,\;\ldots ,\;K\right\}$$

where $M_{k}$ is the number of pattern neurons of class k, and $w_{ki}$ are positive coefficients satisfying, $\overset{M_{k}}{\underset{i=1}{\sum }}w_{ki}=1$. Finally, the *output* (or decision) layer unit classifies the pattern vector x in accordance with the Bayes’s decision rule based on the output of all the *summation* layer neurons:

(5) $$C\left(x\right)=\arg\underset{1\leq k\leq K}{\max}\left(p_{k}\right)$$

It is worth mentioning that the smoothing parameter (σ) is the only parameter of the network that needs to be fixed at the beginning of the training process.

### 4.2. Clustering Based Prediction

By running a MLE per sub-area, as described in the previous subsection, we can provide recommendations to mobile operators about which specific sub-areas are going to be more crowded, in order to plan the activation or deactivation of the corresponding datacells. However, the execution of the above mentioned process for each separate sub-area might consume a lot of resources, as well as time.

In order to reduce this complexity, we propose the use of a clustering based prediction mechanism. Specifically, we propose the formulation of clusters of sub-areas (*i.e.*, group-areas), where users’ habits present similar characteristics. By doing so, we can minimize the number of MLEs that need to be created for each centralized network controller and at the same time reduce the complexity of the overall process. The clustering based prediction consists of the following two steps:

1. *Clustering*: As a first step, we cluster the available datasets for n sub-areas into k clusters (k<n) using a clustering algorithm, described in the next subsection.
2. *Classification*: The data from each cluster (1,…,k), are used for classification employing one of the prediction algorithms that are analysed in Section 4.1.

For the purposes of this paper, we use a K-means clustering algorithm in order to create clusters of the available sub-areas [32].

#### K-Means Clustering

K-Means is one of the most popular clustering methods, which can be defined as the partitioning of a finite amount of data into a number of clusters by understanding the underlying structure [32]. It is noted, that this problem belongs to the general class of NP-hard problems, and as a result, several heuristic algorithms are commonly employed for convergence to an optimum solution.

Two important issues in using the K-means clustering algorithm is the determination of the optimal number of clusters and the centre of each cluster. However, given the number of clusters, the problem is reduced in finding the centre of the clusters. As a result, assuming that there are n observations $\left(x_{1},x_{2},\ldots ,x_{n}\right)$, and k sets $S=\;\left(S_{1},S_{2},\ldots ,S_{k}\right)$, the objective function is:

(6) $$\arg\underset{S}{\min}\overset{k}{\underset{i=1}{\sum }}\underset{x\in S_{i}}{\sum }{\left\|x-\mu_{i}\right\|}^{2}$$

where $\mu_{i}$ is the mean of $S_{i}$.

**Figure 4:**
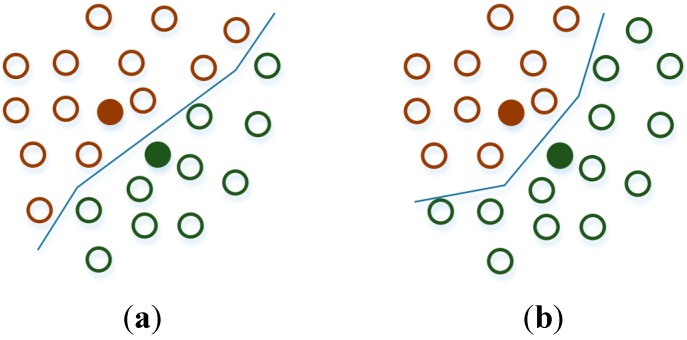
K-Means Clustering example with 2 clusters. (**a**) Initial clusters; (**b**) Final clusters.

A simple example of using K-means with two clusters is given in Figure 4. At the initial stage, the data are divided into two non-optimal clusters, while at the final stage the clusters have reached their ending form.

### 5. Data Analysis

**Figure 5:**
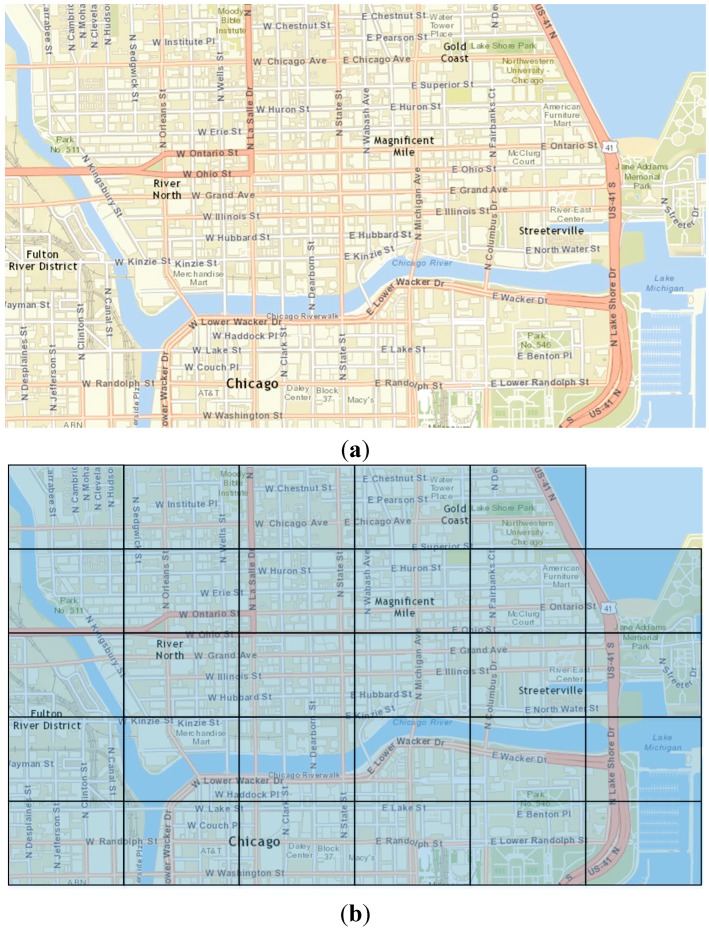
(**a**) Map of Chicago area, used for collecting data from Social Network; (**b**) Map of Chicago area divided into sub-areas.

The dataset used in this paper is based on measurements performed for research purposes in [33]. The data were collected from a well-known social network (Foursquare), which allows location-based check-ins, as well as ratings. The available data refer to the geographical limits of a certain area in Chicago, forming the map as shown in Figure 5a. In addition, the map was divided into 29 sub-areas, where each area may contain more than one BSs (Figure 5b).

**Figure 6:**
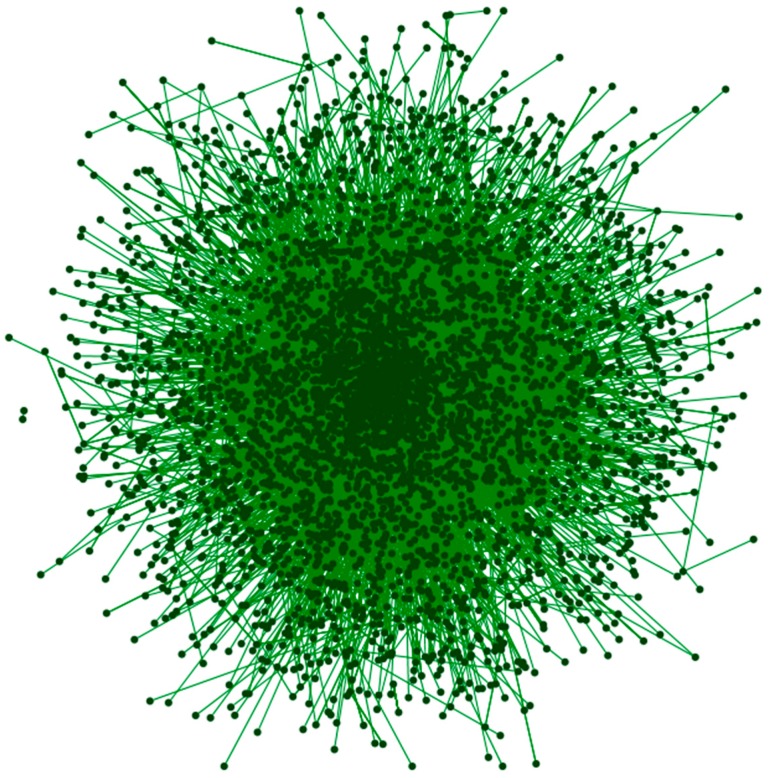
Social graph using Fruchterman-Reingold lay out algorithm s.

The information extracted from the specific social network includes each user’s check-ins for the period of one year, as well as his friendship status with other members of the same social network. The friendship connection of each user results to a social graph depicted in Figure 6. From the friendship connections, we create two innovative variables, namely *influencers* and *influencees*, according to the number of friends each user has influenced in order to visit a specific area or he has been influenced to visit this area, correspondingly.

The above mentioned information is used to create a seven-tuple with the variables described in Table 1.

**Table 1:** Variables and values used.

Variable	Description
sub-area	Sub-area of the created map {29 sub-areas}
day	Values 1–7, representing each day of the week {Monday–Sunday}
month	Values 1–12, representing each moth {January–December}
event	A binary variable representing any special event that may occur in each specific day (e.g., Christmas, Easter, *etc*.)
period	Values 1–4, representing the time period of each day {morning, noon, afternoon, night}
influencers	The number of friends that the user has influenced to visit a sub-area at the specific time
influencees	The number of friends that have influenced the user to visit a sub-area at the specific time

Using the described variables, the input set for the proposed algorithms can be defined as:

(7) x= (subarea, day,month,event,period, influencers,influencees)

In order to provide mobile operators with recommendations about whether a sub-area will be crowded or not, we use the methods described in Section 4. Furthermore, we introduce as a target for the used prediction algorithms namely the crowd_level variable, which takes values from 1 to 3 according to the level of forecasted crowd {uncrowded, crowded, overcrowded}.

### 6. Results

**Figure 7:**
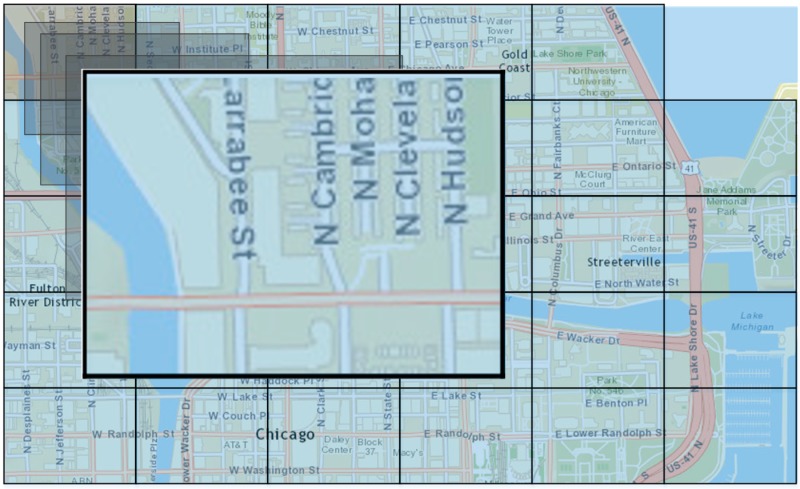
Map of Chicago—*subarea-1*.

To demonstrate the appropriateness of the proposed models, presented in Section 4, we compare the results of the validation process of the PNN (σ=0.12), with the MLP and the SVM algorithms. The proposed learning algorithms were used in order to provide recommendations to mobile operators based on users’ check-ins, about the level of crowds expected in one of the investigated sub-areas (*i.e.*, *subarea-1*) during the *noon* (Figure 7). The input set for the proposed algorithms can now be defined as:

(8) x= ( day,month,event, influencers,influencees)

In Table 2, we present the misclassification percentage that was derived from each learning method, using 10-fold cross-validation method. Specifically, the MLP was formulated as a 5-8-3 three-layered feed-forward neural network, while the SVM was constructed with an RBF kernel function.

**Table 2:** Results of validation process.

Learning Method	Misclassification Percentage (%)
3-layered NN	9.667
SVM	6.452
PNN (σ=0.12)	5.645

**Figure 8:**
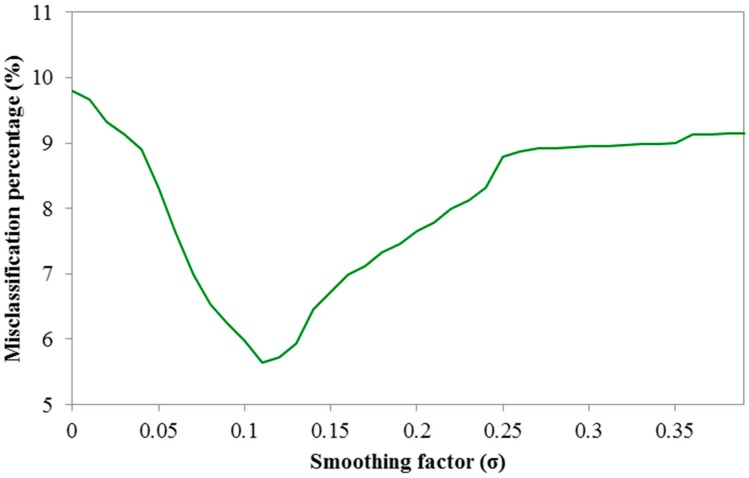
Misclassification percentage for PNN with respect to σ values.

It is clear that the PNN network outperforms both the three-layered perceptron network and the SVM, making it highly suitable for location recommendations. However, it should be noted that the smoothing parameter (σ) affects the performance of the PNN and needs to be appropriately determined in order to give better prediction results. In Figure 8, we present the effect of the smoothing parameter with values that range from 0.01 to 0.4. It can be observed that for all possible σ values, the PNN network in most cases performs better that the other two prediction algorithms, and for σ≈0.12 it provides minimum misclassification percentage.

To further clarify the appropriateness of the proposed learning algorithms, we further study their behaviour by providing predictions per sub-area, as well as per cluster in the following subsections.

### 6.1. Prediction per Sub-Area

As described in Section 4, in order to decide how many datacells will be active, we separate each geographical area covered by the *Centralized Network Controller* into sub-areas, with each sub-area possibly consisting of more than one BSs. For each sub-area, we create a separate MLE, in which we apply a prediction model for the number of active users. We use three different prediction algorithms based on machine learning, namely MLP, SVM and PNN. For the PNN, the σ values minimizing the misclassification percentage are selected for each separate running of the learning algorithm. Similarly, the form of each MLP that is created differs, while SVMs were constructed with an RBF kernel function. The input set for the proposed algorithms is described by Equation (8).

**Figure 9:**
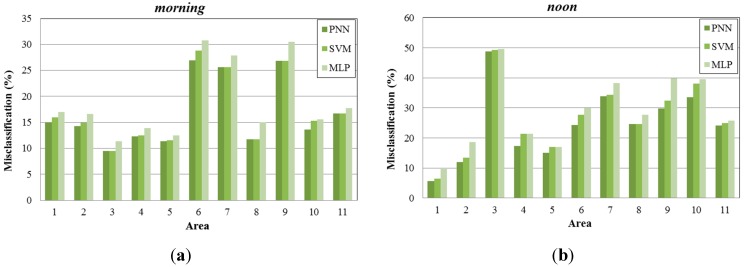

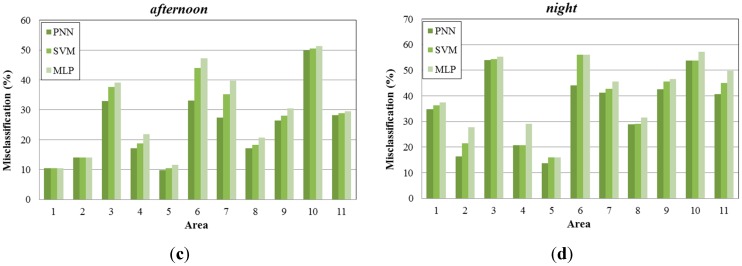
Misclassification percentage per sub-area, (**a**) during morning periods; (**b**) during noon periods; (**c**) during afternoon periods; (**d**) during night periods.

In Figure 9, we present the misclassification percentage gained by each learning algorithm examined (PNN, SVM, MLP) for 11 sub-areas of the created map (Figure 5b), under four different time periods (*morning, noon, afternoon, night*). As observed, PNN provides lower misclassification percentage compared to the other two methods. MLP produces higher misclassifications, while SVM performs better and in some occasions the misclassification percentage is similar to the one produced by PNN.

### 6.2. Clustering Based Prediction

In order to further improve the process of providing recommendations to mobile operators about the activation and deactivation of datacells, we propose the clustering of the available sub-areas to group-areas that include sub-areas with similar users’ characteristics. As described in Section 4, the clustering based prediction consists of two steps. We begin with the clustering of the available areas and then we proceed with the execution of the learning algorithms per cluster.

#### 6.2.1. Clustering

One important parameter that needs to be set in the K-means algorithm is the number of clusters that the dataset is going to be divided into. In order to choose the appropriate number of clusters, we use the gap statistic based on [34]. For the calculation of the gap function, the authors in [34] proposed to standardize the graph of $\log\left(W_{k}\right)$ by comparing it with its expectation under an appropriate null reference distribution of the data, where $W_{k}$ is the pooled within-cluster dispersion measurement:

(9) $$W_{k}=\;\overset{k}{\underset{r=1}{\sum }}\frac{1}{2n_{r}}D_{r}$$

where n is the sample size, $n_{r}$ is the number of data points in cluster r, k is the number of clusters being evaluated, and $D_{r}$ is the sum of the pairwise distances for all points in cluster r.

The gap value is defined as:

(10) $$Gap_{n}\left(k\right)=\;E_{n}^{*}\left\{\log\left(W_{k}\right)\right\}-\log\left(W_{k}\right)$$

where $E_{n}^{*}$ denotes expectation under a sample of size n from the reference distribution. The expected value $E_{n}^{*}\left\{\log\left(W_{k}\right)\right\}$ is determined by Monte Carlo sampling from a reference distribution, and $\log\left(W_{k}\right)$ is computed from the sample data.

**Figure 10:**
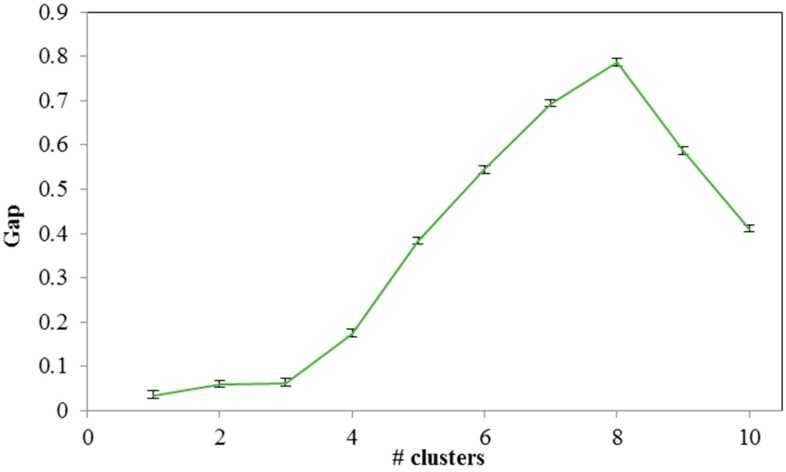
Gap analysis for clustering sub-areas.

Under the gap criterion, the optimal number of clusters occurs at the solution with the largest local or global gap value within a tolerance range. In Figure 10, we present the calculated gap values per suggested number of clusters. As we can see, the proper number of clusters is close to eight, where the maximum value of the gap criterion occurs.

#### 6.2.2. Prediction per Cluster

**Figure 11:**
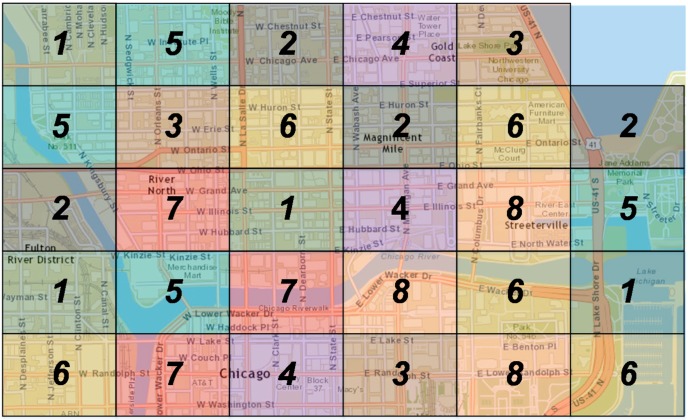
Map of Chicago area divided into clusters for the night period.

Using the previously discussed process, we cluster the available sub-areas into eight clusters. The clustering takes place for each different time period of the day, since users’ mobility varies from one time period to another. For example, in Figure 11 we may observe the formulation of the clusters for the night time period. Each cluster is depicted with a different colour, while it contains more than one sub-areas. It is worth mentioning that the formulation of the clusters varies for each different time period.

Similarly to the prediction per sub-area, we use the three aforementioned prediction algorithms based on machine learning, namely MLP, SVM and PNN. For the PNN, the σ values minimizing the misclassification percentage are selected for each separate running of the learning algorithm. In addition, the form of each MLP that is created differs, while SVMs were constructed with an RBF kernel function. The input set for the proposed algorithms can now be defined as:

(11) x= ( subarea,day,month,event, influencers,influencees)

**Figure 12:**
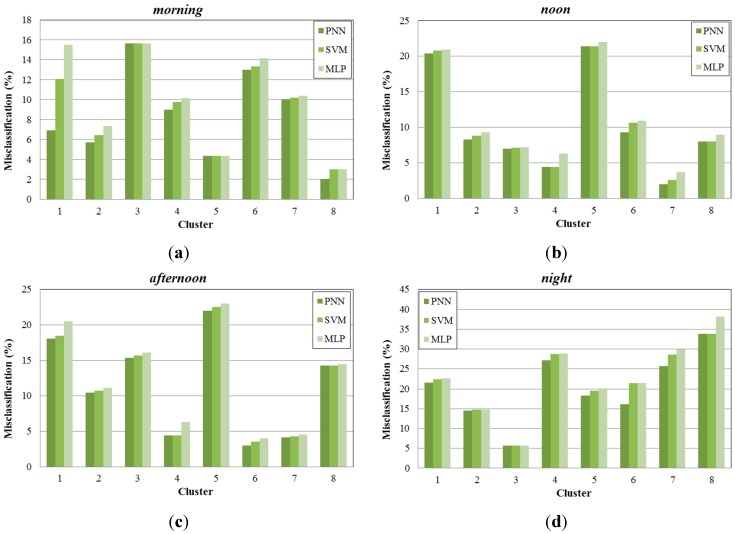
Misclassification percentage per cluster, (**a**) during morning periods; (**b**) during noon periods; (**c**) during afternoon periods; (**d**) during night periods.

In Figure 12, we present the misclassification percentage gained by each learning algorithm examined (PNN, SVM, MLP) for the eight created clusters, under four different time periods (*morning, noon, afternoon, night*). As we can see, PNN provides more accurate results than the other two methods, achieving lower misclassification percentage values, compared to the case of the per area prediction method. Specifically the average reduction of the misclassification percentage for the PNN reaches the 50.25%, 58.70%, 52.71% and 42.68% for morning, noon, afternoon and night time periods respectively. This is explained by the clustering performed in the sub-areas, which resulted to the creation of data with similar characteristics.

## 7. Use Cases

In this section, we present two indicative use case scenarios in order to demonstrate how operators can benefit from the proposed methodology.

### 7.1. Uninhabited Areas

One of the biggest challenges that mobile operators will have to face, during the development of smart cities, is the coverage of large areas in order to be able to support the interconnection of the expected smart objects. However, this process is extremely energy consuming, since BSs need to be always active, regardless the existing traffic. For example, most large cities have amusement parks where people usually visit only for pleasure and usually only on weekends. Therefore, the continuous coverage of the entire area is energy consuming and also expensive for the operators. Using our proposed system, we can extract knowledge from social networks and estimate the time period that the specific area will be crowded. With this knowledge, operators can schedule to activate more cells in uninhabited areas for the periods that are characterized as crowded, while they can deactivate the majoring of the available cells for “dead” periods, in order to be more energy efficient and cost-efficient.

### 7.2. Residential Areas

In residential areas, in order to provide services to users and maintain their satisfaction at a high level, operators need to install cells according to the number of users served in each area. However, some areas are more crowded during specific periods or days. For example, there are areas providing more an abundance of entertainment options that gather more people during night. Although these areas in small cities are usually predictable, in large cities they might change from time to time following current trends, thus forming new trending areas. Using our proposed architecture, we can identify these newly trending areas from location based social networks (LBSNs), and specifically from users’ check-ins to points-of-interest (POIs). In that way, operators can schedule the activation and deactivation of cells during the daytime, according to people habits.

## 8. Conclusions

In this paper, we presented our vision on enhancing next generation wireless networks with knowledge that is extracted from SNs. We have discussed a novel approach for connecting software-based controllers that rely on SDNs, to OSNs. An overview of the deployment architecture was presented, while the foreseen components of the identified architecture and their interactions were conferred in detail.

Three different learning algorithms, namely MLP, SVM and PNN, were used in order to predict the crowd level in specific sub-areas. Recognizing the complexity of running a separate machine learning engine per sub-area, we proposed a clustering based prediction model, where the available data were clustered using K-means clustering algorithm.

The dataset used includes information collected from a well-known social network (Foursquare), depicting users’ friendships. From the results acquired, we have concluded that the PNN outperforms the other two proposed algorithms, giving predictions with higher accuracy, while the clustering of the sub-areas further enhances this behaviour.

Furthermore, focus was given on data related to users’ check-ins. Apart from using social networks with information about users’ location, like Foursquare, other kinds of social networks can also be exploited. For example, we can extract knowledge from social networks, where event organisers are posting forthcoming events and people declare their possible presence. Following this reasoning, future work could exploit information gathered from other such well-known social networks, like Facebook and Twitter, in order to further enhance the recommendations provided to mobile operators about possible crowded areas.
